# Pioglitazone Hydrochloride Extends the Lifespan of *Caenorhabditis elegans* by Activating DAF-16/FOXO- and SKN-1/NRF2-Related Signaling Pathways

**DOI:** 10.1155/2022/8496063

**Published:** 2022-05-29

**Authors:** Wenjuan Jia, Chongyang Wang, Jingming Zheng, Yimin Li, Caixian Yang, Qin-Li Wan, Jie Shen

**Affiliations:** ^1^Department of Endocrinology and Metabolism, The Third Affiliated Hospital, Southern Medical University, Guangzhou 510630, China; ^2^Department of Endocrinology, Sixth Affiliated Hospital of Guangzhou Medical University, Qingyuan People's Hospital, Qingyuan 511518, China; ^3^Zhuhai Precision Medical Center, Zhuhai People's Hospital (Zhuhai Hospital Affiliated with Jinan University), Jinan University, Guangzhou 510632, China; ^4^Department of Pathogen Biology, School of Medicine, Jinan University, Guangzhou 510632, China; ^5^Institute and Department of Endocrinology and Metabolism, Shunde Hospital, Southern Medical University (The First People's Hospital of Shunde), Foshan 528399, China

## Abstract

Pioglitazone hydrochloride (PGZ), a nuclear receptor peroxisome proliferator-activated receptor gamma (PPAR-*γ*) agonist, is a universally adopted oral agent for the treatment of type 2 diabetes (T2D). Previous studies reported that PGZ could ameliorate the symptoms of aging-related diseases and Alzheimer's disease. However, whether PGZ participates in aging regulation and the underlying mechanism remain undetermined. Here, we found that PGZ significantly prolonged the lifespan and healthspan of *Caenorhabditis elegans* (*C*. *elegans*). We found that a variety of age-related pathways and age-related genes are required for PGZ-induced lifespan extension. The transcription factors DAF-16/FOXO, HSF-1, and SKN-1/NRF2, as well as the nuclear receptors DAF-12 and NHR-49, all functioned in the survival advantage conferred by PGZ. Moreover, our results demonstrated that PGZ induced lifespan extension through the inhibition of insulin/insulin-like signaling (IIS) and reproductive signaling pathways, as well as the activation of dietary restriction- (DR-) related pathways. Additionally, our results also indicated that beneficial longevity mediated by PGZ is linked to its antioxidative activity. Our research may provide a basis for further research on PGZ, as an anti-T2D drug, to interfere with aging and reduce the incidence of age-related diseases in diabetic patients.

## 1. Introduction

Aging is an inevitable biological process that is often accompanied by gradual disorders of the body's homeostasis, a decrease in physiological function, an increase in the risk of disease, and an increase in mortality [1]. In humans and most model organisms, aging is always accompanied by a higher incidence of some diseases, such as cancer, hypertension, type 2 diabetes, Alzheimer's disease, and Parkinson's disease, which lead to large social and economic burdens [1]. Given the urgent need for interventions to promote healthy aging, drugs that can lengthen lifespan and healthspan present a high putative value. To date, several drugs have been identified as having a potential antiaging effect, including pharmaceutical drugs (i.e., metformin [2] and aspirin [3]), natural molecules (i.e., resveratrol [4] and urolithin A [5]), and synthetic compounds (i.e., JZL184 [6] and TES991 [7]). However, pharmaceutical drugs, due to their long-term clinical application, have relatively few and well-known side effects; therefore, they have great value in the development of potential antiaging drugs.

Pioglitazone hydrochloride (PGZ), which is a peroxisome proliferator-activated receptor gamma (PPAR-*γ*) agonist, is widely used as an oral drug for the treatment of hyperglycemia in type 2 diabetes. As an antidiabetic drug that has been identified to have antiaging effects, one of the most well-known drugs is metformin [8], in addition to acarbose [9]. However, the question of whether, and if so how, PGZ as an antidiabetic drug plays a role in lifespan extension remains unanswered. Recently, a study in animal models showed that PGZ can ameliorate learning and memory impairment in a mouse Alzheimer's disease model [10], suggesting that PGZ may have potential antiaging effects. Another study in *Drosophila* reported that PGZ had antiaging properties [11]. However, there has not been an in-depth investigation into the underlying molecular mechanism by which PGZ confers a survival advantage in a published study.

In this study, we used *Caenorhabditis elegans* (*C*. *elegans*) as a model to investigate the mechanism of delaying aging induced by PGZ. The free-living soil nematode *C*. *elegans* is an excellent and robust model organism for studying the mechanism of aging due to its short lifespan, genetic tractability, and conserved developmental programs. Most importantly, aging studies in *C*. *elegans* have provided a wealth of information about the mechanisms of longevity regulation, such as the insulin/insulin-like signaling (IIS) pathway, dietary restriction (DR), mitochondrial function, autophagy, and the unfolded protein response (UPR), which are evolutionally conserved among different species, from yeast to vertebrates [12, 13]. In this work, we found that PGZ significantly prolonged the lifespan and healthspan of *C*. *elegans* through inhibition of IIS and the reproductive signaling pathway and activation of the DR-like signaling pathway in an AMPK-dependent manner, thereby activating the transcription factors DAF-16/FOXO, HSF-1, and SKN-1/NRF2, as well as the nuclear receptors DAF-12 and NHR-49.

## 2. Results

### 2.1. PGZ Lengthens the Lifespan and Healthspan of *C*. *elegans*

**Figure 1:**
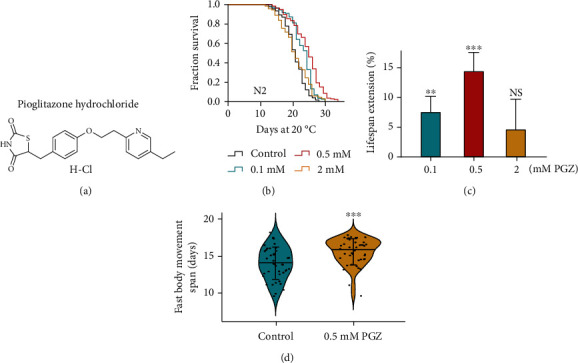
PGZ extends the lifespan of *C*. *elegans*. (a) Chemical structure of PGZ. (b) Survival curves of wild-type N2 worms exposed to increasing concentrations of PGZ (0.1-2 mM). (c) Dose-dependent analyses of the effect of PGZ on the lifespan of *C*. *elegans*. Error bars represent the SD. Mean ± SD of the percentage of lifespan extension from independent experiments (means ± SD; ^∗∗∗^*p* < 0.001, ^∗∗^*p* < 0.01, and NS (not significant); Student's *t*-test). Lifespan was calculated with the Kaplan-Meier test, and *p* values were calculated using the log-rank test. Experiments were repeated at least twice, and detailed lifespan values were summarized in Supplementary Table S1. (d) Age-related movements of worms treated with 0.5 mM PGZ and those of untreated controls (means ± SD; *n* ≥ 30; ^∗∗∗^*p* < 0.05; Student's *t*-test).

To investigate whether PGZ (Figure 1(a)) plays a role in lifespan regulation, we treated adult worms with various concentrations of PGZ and conducted survival analyses. Our results showed that diverse concentrations of PGZ were capable of increasing the lifespan of *C*. *elegans* (Figures 1(b) and 1(c)), while 0.5 mM PGZ exhibited the greatest and most robust lifespan extension effect (Figures 1(b) and 1(c)). Therefore, 0.5 mM was the concentration used in all subsequent experiments.

Lifespan extension is not always related to vitality. To assess whether PGZ also promotes the healthspan of *C*. *elegans*, we detected the effect of PGZ on body movement, which was characterized as a parameter associated with aging [14]. We found that PGZ could significantly extend the period of fast movement of worms by 17.42%, suggesting that PGZ could also lengthen the healthspan of *C*. *elegans* (Figure 1(d)). According to the above results, we concluded that PGZ significantly prolonged the lifespan and healthspan of *C*. *elegans*, with the concentration of 0.5 mM displaying the greatest extension effect.

### 2.2. PGZ-Induced Lifespan Extension Depends on the IIS Pathway

**Figure 2:**
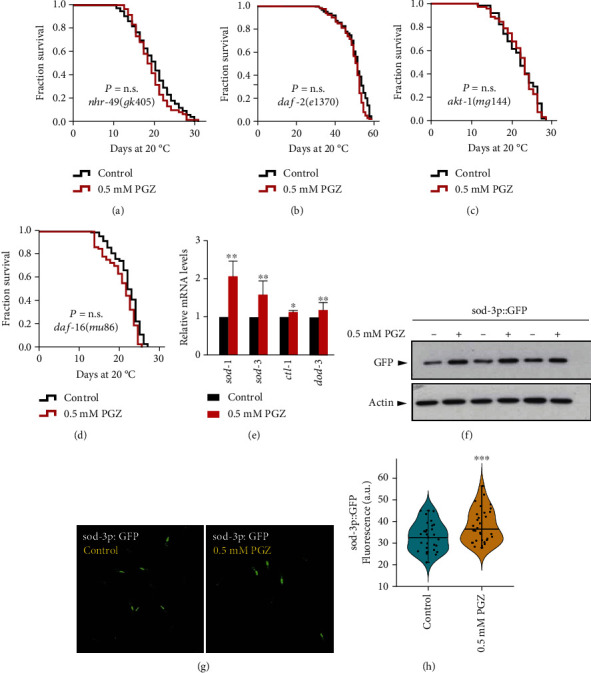
The effect of PGZ on lifespan extension depends on the IIS pathway. (a–d) Lifespan analyses of *nhr*-*49(gk405)* (a), *daf*-*2(e1370)* (b), *akt*-*1(mg144)* (c), and *daf*-*16(mu86)* (d) mutant worms treated with or without 0.5 mM PGZ (*p* value by the log-rank test). (e) mRNA levels of *daf*-*16* target genes (*sod*-*1*, *sod*-*3*, *ctl*-*1*, and *dod*-*3*) in WT animals with or without PGZ treatment (means ± SD; *n* = 3; ^∗^*p* < 0.05 and ^∗∗^*p* < 0.01; Student's *t*-test). (f) Western blot analyses of SOD-3p::GFP in worms treated with or without 0.5 mM PGZ. Actin was shown as the loading control. (g) Imaging of fluorescence in the transgenic strain CF1553 (SOD-3p::GFP) (representative of three experiments) and (h) respective quantitative results for at least 30 worms per condition (means ± SD; *n* ≥ 30; ^∗∗∗^*p* < 0.001; Student's *t*-test).

We next asked whether any and which commonly known aging pathways mediated lifespan extension induced by PGZ. Since PGZ is a peroxisome proliferator-activated receptor gamma (PPAR-*γ*) agonist, nuclear receptor PPAR*α* homolog NHR-49 may be important for its lifespan-extending effects [15]. We first determined the role of NHR-49 in PGZ-induced lifespan extension. The results showed that impairment of NHR-49 abolished the lifespan extension induced by PGZ, suggesting that NHR-49 is necessary for the longevity-promoting effect of PGZ (Figure 2(a)).

Considering PGZ is the most common drug for T2D, we investigated the role of the IIS pathway, which is well known to regulate lifespan in different species [12]. We analyzed the roles of *daf*-*2* (a well-known insulin-like receptor) and *akt*-*1* (a kinase downstream of *daf*-*2*) in the survival advantage induced by PGZ. The results showed that treatment with 0.5 mM PGZ failed to further increase the lifespan of the *daf*-*2* and *akt*-*1* mutants (Figures 2(b) and 2(c)). In *C*. *elegans*, the insulin receptor DAF-2 regulates the activity of PI3K/AGE-1 and then activates the downstream kinases, PDK-1 and AKT-1/2, to control the transcription factor DAF-16/FOXO, thereby regulating longevity [16, 17]. Similar to the results of *daf*-*2* and *akt*-*1* mutants, *daf*-*16* mutants were also unresponsive to PGZ (Figure 2(d)). Moreover, we also found that PGZ treatment increased the endogenous mRNA levels of DAF-16 target genes, including *sod*-*1*, *sod*-*3*, *ctl*-*1*, and *dod*-*3* [16] (Figure 2(e)). In addition, SOD-3 expression levels were shown to be increased using a worm strain expressing SOD-3p::GFP (Figures 2(f)–2(h)). Consequently, these results illustrated that the longevity-promoting effect of PGZ might be mediated by inhibiting the IIS pathway, subsequently activating the transcription factor DAF-16 and promoting the transcription of its target genes.

### 2.3. The Effect of PGZ on Lifespan Extension Was Conferred by the Transcription Factors HSF-1 and SKN-1

**Figure 3:**
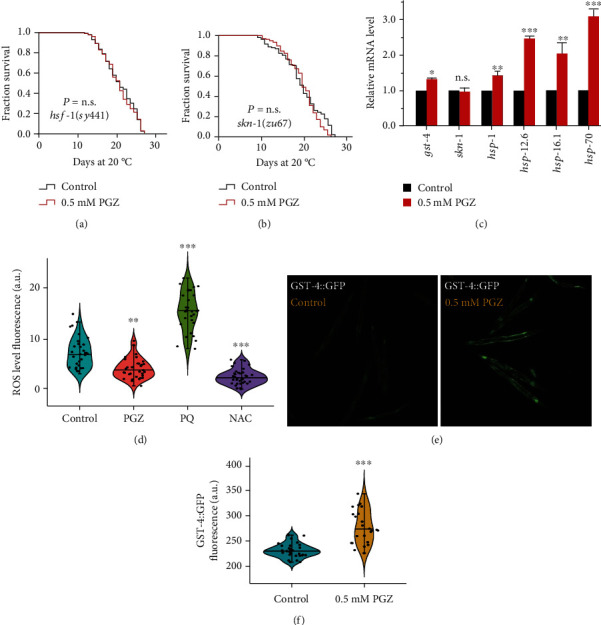
The effect of PGZ on lifespan extension depends on the transcription factors SKN-1/NRF2 and HSF-1. (a, b) Survival analyses of *hsf*-*1(sy441)* (a) and *skn*-*1(zu67)* (b) mutants exposed to 0.5 mM PGZ compared with the untreated control. (c) qPCR analyses of the mRNA levels of the target genes of *hsf*-*1* (*hsp*-*1*, *hsp*-*12*.*6*, *hsp*-*16*.*1*, and *hsp*-*70*) and *skn*-*1* (*gst*-*4*). (d) Quantitation of intracellular levels of ROS in animals treated with PGZ, PQ, and NAC and nontreated controls. PQ represents paraquat, and NAC is the abbreviation for N-acetyl cysteine. (e, f) Image and quantitation of GFP fluorescence in the transgenic strain CL2166 (GST-4p::GFP). Data were the means ± SD; *n* ≥ 30; ^∗∗^*p* < 0.01 and ^∗∗∗^*p* < 0.001; Student's *t*-test.

In addition to DAF-16, the well-known and crucial longevity transcription factors acting downstream of the IIS pathway also include HSF-1 and SKN-1 [18, 19]. To determine whether PGZ regulated the IIS pathway in an HSF-1-, SKN-1-, or both-dependent manner, we tested the effects of 0.5 mM PGZ on *hsf*-*1(sy441)* and *skn*-*1(zu67)* mutants. The results showed that, similar to that of *daf*-*16* mutants, the survival advantages induced by PGZ were abrogated in *hsf*-*1(sy441)* (Figure 3(a)) and *skn*-*1(zu67)* mutants (Figure 3(b)). Moreover, the mRNA levels of the target genes of SKN-1 (*gst*-*4*) [20] and HSF-1 (*hsp*-*1*, *hsp*-*12*.*6*, *hsp*-*16*.*1*, and *hsp*-*70*) [19] were significantly elevated in the WT animals treated with PGZ compared with the nontreatment control (Figure 3(c)). Additionally, we also detected that the GFP fluorescence intensity of transgenic strain GST-4::GFP was considerably elevated when animals were exposed to 0.5 mM PGZ compared with the nontreatment control (Figures 3(e) and 3(f)). Altogether, these results demonstrated that the transcription factors SKN-1 and HSF-1 contributed to the survival advantage caused by PGZ, which might act downstream of *daf*-*2*.

SKN-1, an NRF2 ortholog, is a key oxidative stress response transcription factor that activates antioxidant and phase II detoxification genes to regulate longevity [21]. DAF-16 is a central transcription factor that responds to different types of stress, such as inducing the expression of gene-related oxidative stress (i.e., superoxide dismutase (SOD)) to achieve resistance to oxidative stress [16]. Given the involvement of the transcription factors SKN-1 and DAF-16 in the longevity effects of PGZ, we asked whether the longevity effect caused by PGZ is linked to antioxidant activity. We measured the intracellular ROS accumulation levels using 2′,7′-dichlorodihydrofluorescein diacetate (H2DCF-DA), a free radical sensor that is deacetylated by intracellular esterases that then emits detectable fluorescence signals to present the level of intracellular ROS [22]. Our results showed that PGZ treatment obviously decreased the level of ROS compared with the nontreatment control (Figure 3(d)). Supporting the result of ROS reduction, our previous results showed that PGZ upregulated the expression levels of *sod*-*1* and *sod*-*3*, which are members of an important class of antioxidant genes, superoxide dismutases, which convert superoxide into hydrogen peroxide. Accordingly, these results indicated that lifespan extension induced by PGZ might be associated with its antioxidative activity.

### 2.4. PGZ Extends the Lifespan of *C*. *elegans* by Regulating DR-Like and Reproductive Signaling Pathways

**Figure 4:**
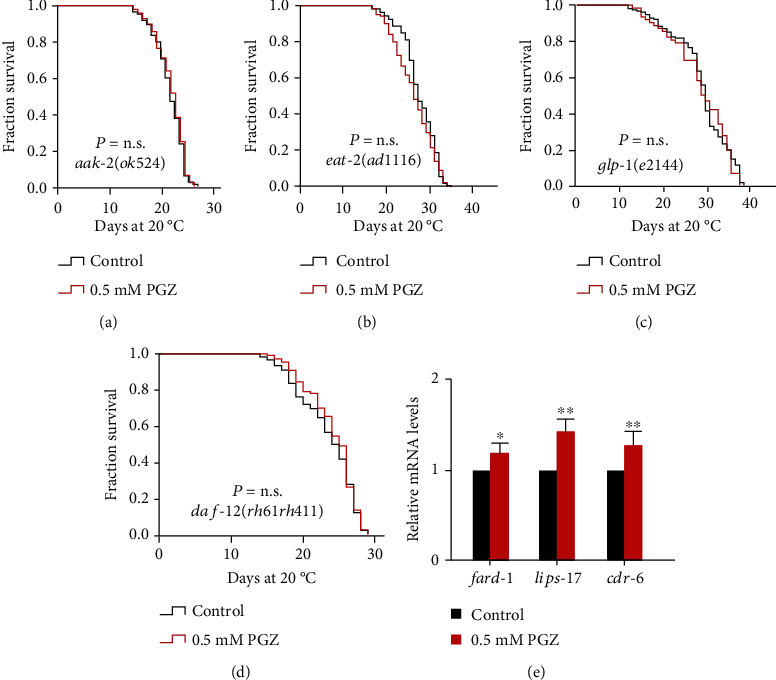
PGZ-induced lifespan extension depends on its regulation of DR-like and reproductive signaling pathways. (a–d) Survival analyses of *aak*-*2(ok524)* (a), *eat*-*2(ad1116)* (b), *glp*-*1(e2144)* (c), and *daf*-*12(rh61rh411)* (d) mutants treated with or without 0.5 mM PGZ (*p* value by the log-rank test). The lifespan values of the repeat experiments were summarized in Supplementary Table S1. (e) mRNA expression levels of *daf*-*12* target genes (*fard*-*1*, *lips*-*17*, and *cdr*-*6*) (means ± SD; *n* = 3 independent experiments; ^∗^*p* < 0.05 and ^∗∗^*p* < 0.01; Student's *t*-test).

Lifespan extension induced by PGZ is dependent on DAF-16, a central longevity regulator that is downstream of several kinases, such as AMP-activated kinase (AMPK), in addition to the aforementioned AKT-1 in the IIS pathway. It has been reported that in *C*. *elegans*, the constitutive activation of AMPK increases animal longevity and stress resistance in a DAF-16-dependent manner [23]. AMPK is also a therapeutic target of the well-known antidiabetic drug metformin [24]. We thus detected whether PGZ activates DAF-16 to extend the lifespan due to the induction of AMPK activity. We found that the longevity effect induced by PGZ was blocked with the loss of function of *aak*-*2*, a homolog of the catalytic *α* subunit of AMPK (AMPKa2) in *C*. *elegans* [25] (Figure 4(a)).

In *C*. *elegans*, AMPK is a well-known central energy metabolism regulator that is activated under low-energy conditions and is necessary for lifespan extension caused by DR [17, 26]. DR is a conservative life-prolonging intervention that can regulate the expression of a series of antioxidant enzyme genes, thereby prolonging lifespan [27]. Both AMPK and DAF-16 respond to DR-induced longevity, reminding us that DR-related mechanisms also play a role in PGZ-induced lifespan extension. Therefore, we determined the effect of PGZ on the lifespan of the *eat*-*2* mutant, which is a model that simulates DR by reducing food intake due to damage to the pharyngeal pump. Indeed, we found that impairment of *eat*-*2* expression completely abrogated the survival advantage induced by PGZ (Figure 4(b)), suggesting that PGZ might, at least in part, promote an increased lifespan by regulating DR-related signaling pathways.

In addition to insulin and DR-related signaling pathways, the activation of NHR-49, DAF-16, and SKN-1 by PGZ is reminiscent of the mechanism regulated in the reproductive signaling pathway. In *C*. *elegans*, it has been reported that removing the germline can significantly lengthen lifespan by approximately 60% by regulating the transcription factors NHR-49, DAF-16, and SKN-1, at least in part [28, 29]. We assessed the effect of PGZ on the long-lived *glp*-*1(e2144)* mutant, a temperature-sensitive mutant that is long-lived when maintained at nonpermissive temperatures due to failed germline proliferation. Our results showed that PGZ failed to further extend the lifespan of *glp*-*1* mutants (Figure 4(c)). Furthermore, we also found that the longevity benefit of PGZ was abolished in short-lived *daf*-*12(rh61rh411)* mutants (Figure 4(d)). DAF-12, a nuclear steroid receptor, is a crucial regulator downstream of the reproductive signaling pathway and functions in lifespan regulation caused by germline loss in *C*. *elegans* [30]. Likewise, we detected a significant increase in the expression of the target genes of DAF-12 (*fard*-*1*, *lips*-*17*, and *cdr*-*6*) [31] when WT worms were exposed to 0.5 mM PGZ compared with the control (Figure 4(e)). In summary, these findings illustrated that the survival advantage of PGZ is mediated through regulating the reproductive signaling pathway in an NHR-49-, DAF-16-, SKN-1-, and DAF-12-dependent manner.

### 2.5. A Mitochondrial Stress Response May Be Dispensable for the Survival Advantage Induced by PGZ

**Figure 5:**
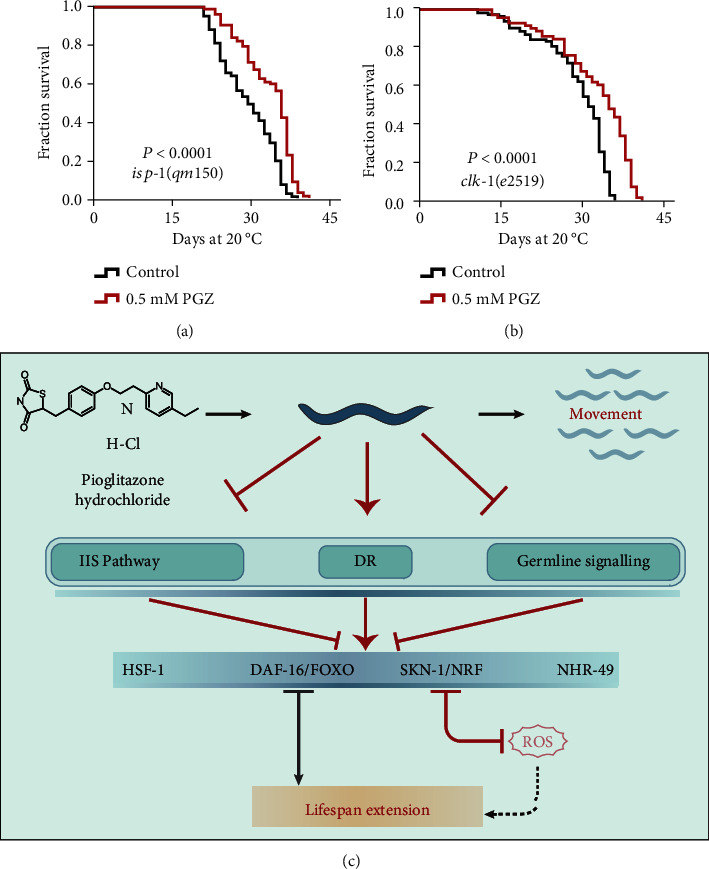
The effect of PGZ on lifespan extension is independent of mitochondrial function. (a, b) Survival analyses of *isp*-*1(qm150)* (a) and *clk*-*1(e2519)* (b) mutants exposed or not exposed to 0.5 mM PGZ (*p* value by the log-rank test). The lifespan values of repeat experiments were summarized in Supplementary Table S1. (c) Mechanisms of action of PGZ in *C*. *elegans*.

Given the antioxidant activity of PGZ contributing to its lifespan benefit in *C*. *elegans*, it is well known that ROS are generated as a byproduct of normal metabolism in the mitochondria [32, 33]. Several lines of evidence have demonstrated that inhibition of mitochondrial function leads to lifespan extension due to reduced oxygen consumption and ROS generation [34]. These findings motivated us to question whether mitochondrial function participates in PGZ-induced lifespan benefits. Using two long-lived mitochondrial dysfunctional mutants, *isp*-*1*, a Rieske iron-sulfur protein of mitochondrial respiratory chain complex III [35], and *clk*-*1*, an ortholog of human COQ7 (coenzyme Q7, hydroxylase) [36], we found that PGZ could extend the lifespan of these mutants (Figures 5(a) and 5(b)), suggesting that a mitochondrial stress response was not necessary for the survival advantage induced by PGZ.

## 3. Materials and Methods

### 3.1. Nematode Strains and Maintenance

*C*. *elegans* strains were maintained on nematode growth medium (NGM) plates with *Escherichia coli* (*E*. *coli*) OP50 at 20°C as previously described [37]. For all experiments, synchronized populations were generated through a standard bleaching protocol. The following strains were used in this study: wild-type N2, CF1038 *daf*-*16(mu86) I*, EU1 *skn*-*1(zu67) IV*, CB1370 *daf*-*2(e1370) III*, PS3551 *hsf*-*1(sy441) I*, CF1903 *glp*-*1(e2144) III*, MQ887 *isp*-*1(qm150) IV*, CB4876 *clk*-*1(e2519) III*, DA1116 *eat*-*2(ad1116) II*, AA86 *daf*-*12(rh61rh411) X*, GR1310 *akt*-*1(mg144) V*, RB754 *aak*-*2(ok524) X*, VC870 *nhr*-*49(gk405) I*, CF1553 muIs84 [(pAD76)sod-3p::GFP+rol-6(su1006)], and CL2166 dvIs19 [(pAF15)gst-4p::GFP::NLS]. The strains used in this study were provided by the *Caenorhabditis* Genetics Center (University of Minnesota), which is supported by the NIH NCRR.

All compounds used in this work were purchased from Sigma-Aldrich (Munich, Germany). PGZ and NAC were dissolved in water. All NGM plates with compounds were equilibrated overnight before use. Before conducting the corresponding experiments, worms were cultured on NGM plates with *E*. *coli* OP50 for 2-3 generations without starvation.

### 3.2. Lifespan Assays

Lifespan assays were performed with a standard protocol as previously described [38]. Briefly, approximately 100 young adult worms were transferred to fresh plates containing the respective concentrations of compounds and 10 *µ*M 5-fluoro-2′-deoxyuridine (FUdR, Sigma) to prevent offspring. For CF1903, a temperature-sensitive mutant, during survival analysis, L1 CF1903 worms were incubated at 20°C for 12 h, transferred to 25°C until young adulthood to eliminate germline development, and then returned to 20°C for the remainder of the lifespan. For survival analyses, heat-inactivated bacteria were used to prevent the metabolism of compounds by bacteria. During survival analyses, the animals were transferred to fresh plates every other day to ensure drug potency. Death events were scored daily. Statistical analyses were carried out using SPSS software. *p* values were calculated by the log-rank test, and *p* < 0.05 was accepted as statistically significant. The experiment was repeated at least three times. The mean, SEM, *p* value, and lifespan value are summarized in Supplementary Table S1.

### 3.3. Period of Fast Body Movement Assays

The period of fast body movement assays was performed as described previously [39]. Briefly, at least 100 young adults were transferred to fresh plates with or without compounds and maintained as described in the lifespan assay. The movement of nematodes was scored daily. When tapping plates, the worms moving in a continuous, coordinated sinusoidal way were characterized as fast movement; otherwise, the worms were defined as a nonfast movement.

### 3.4. Fluorescence Microscopic Imaging

The measurement of the GFP fluorescence intensity of *C*. *elegans* was conducted as previously described [40]. The activities of GST-4::GFP and SOD::GFP were analyzed using transgenic strains CL2166 and CF1553, respectively. For CL2166 and CF1553, synchronized late L4 larvae were treated with or without 0.5 mM PGZ for 12 h. Then, the animals were transferred to 2% agarose pads after paralyzing them using 10 *µ*M levamisole and were imaged using a Nikon Ti2-U epifluorescence microscope with a 20x air objective. Images were analyzed using ImageJ software. More than 30 worms were used for each experiment. Statistical analyses were performed using GraphPad Prism, and the *p* value was calculated using a two-tailed Student's *t*-test.

### 3.5. Detection of Intracellular Reactive Oxygen Species (ROS) Accumulation

The levels of endogenous reactive oxygen species (ROS) were measured using 2′,7 ′-dichlorodihydrofluorescein diacetate (H2DCF-DA) as previously described [41]. Briefly, synchronized L1 larvae were spread onto plates with the respective compound until they reached the young adult stage. Then, the animals were transferred to plates with 10 *µ*M 2′,7′-dichlorodihydrofluorescein diacetate (H2DCF-DA) and cultured for 1 h to visualize endogenous ROS. Here, H2DCF-DA was spread, plated through mixing with live OP50, and maintained at a final concentration of 10 *µ*M. Imaging statistical analyses were performed using the same protocol as used for the fluorescence analyses.

### 3.6. Quantitative RT-PCR Assay

Animals were synchronically raised on plates with or without PGZ at 20°C as described for the lifespan analyses. Total RNA was isolated from young adult-stage worms using RNAiso Plus (Takara) based on the phenol-chloroform extraction method [42]. Afterwards, purified RNA was synthesized into cDNA using a reverse transcription kit (Takara). qRT-PCR experiments were performed using SYBR Select Master Mix (RK21203, ABclonal) on a CFX96 real-time system (Bio-Rad). Data were analyzed with Bio-Rad CFX Manager 3.1 software using the comparative *ΔΔ*Cq method after normalization to the reference gene *cdc*-*42*. *p* values were calculated using a two-tailed Student's *t*-test. The gene-specific primers used in this study are summarized in Supplementary Table S2 (Supplemental files). For each experiment, independent biological triplicates were conducted.

### 3.7. Western Blot Analyses

Animals maintained as described in fluorescence intensity analyses of CF1553 were collected with the M9 buffer. Animals were then lysed in the RIPA buffer by trituration twice using a TissueLyser at 75 Hz for 6 min at 4°C and centrifuged at 10000 g at 4°C. RIPA samples were quantified with a BCA Protein Assay Kit and boiled at 95°C for 5 min. Proteins were separated using SDS-PAGE and transferred to nitrocellulose membranes. The membranes were blocked in milk and then incubated with the primary antibody against GFP (1 : 5000, Roche, 11814460001) or actin (1 : 5000, Sigma, A1978). The primary antibody was visualized using the horseradish peroxidase-conjugated anti-mouse secondary antibody (1 : 2000) and ECL Western Blotting Substrate.

## 4. Discussion

Aging is a complex and irreversible degradation process that occurs in various tissues and organs of the body and can be manifested by factors such as lifespan, sports vitality, and reproductive ability. Accumulating lines of evidence have demonstrated that aging is always accompanied by risks of diverse chronic diseases, including cardiovascular diseases, neurodegenerative disorders, diabetes, and multiple cancers [1, 43, 44]. As the global aged population increased in abundance, it has brought a serious economic burden to society. Therefore, understanding the mechanism of aging and combating its effects, as well as finding antiaging drugs, have become urgent problems to be solved. It has been reported that either pharmacologic or genetic intervention can prolong lifespan and ameliorate aging-related diseases [45]. For example, DR is a well-known antiaging mechanism that can modulate conserved cellular and physiological pathways in different species, from yeast to humans, to alleviate aging and aging-related diseases [43]. However, it is a great challenge for most people to perform DR due to severe hunger and irritability. Therefore, pharmacological interference is undoubtedly a more practical choice to delay aging.

Considering that diabetes is a chronic disease that accompanies aging, some antidiabetic drugs have been developed into potential antiaging drugs, such as metformin and acarbose, and their antiaging effects and potential corresponding molecular mechanisms have been elucidated in model organisms [2, 9]. In this work, we confirmed that another common drug for the treatment of T2D, PGZ, could extend lifespan in a remarkable way. We found that most aging pathways were involved in lifespan extension mediated by PGZ, including IIS signaling, DR-like signaling, and reproductive signaling pathways. Furthermore, we found that several transcription factors, including DAF-16, HSF-1, and SKN-1, contributed to the survival advantage induced by PGZ. Consequently, these observations are puzzling, as they indicate that PGZ triggers many of the well-known longevity genes and pathways (Figure 5(c)).

The administration of metformin, a first-line drug for the treatment of T2D, has been found to have antiaging effects in a variety of model organisms, including *C*. *elegans* and mice [8, 46–48]. In *C*. *elegans*, several different mechanisms have been identified for metformin's lifespan extension effect. For example, metformin may regulate DR-like mechanisms in an AMPK-dependent manner [24]. Furthermore, alteration of bacterial metabolism has been shown to contribute to regulating aging [48]. Other studies have reported that metformin inhibits the mTORC1 pathway to influence lifespan [2, 47]. For the antidiabetic drug PGZ, we found that it has a similar effect to metformin in prolonging the lifespan of *C*. *elegans* and a similar aging regulation mechanism (i.e., AMPK); however, we also found other mechanisms distinct from metformin regulation, such as the IIS and reproductive signaling pathways. Moreover, we also observed the antioxidative activity of PGZ. To support our findings, previous studies reported that lifespan extension due to inhibition of IIS and activation of AMPK was associated with reducing ROS levels [49, 50]. Likewise, researchers found that DAF-16 acts as a central regulator that receives signals upstream, such as DAF-2 and AMPK, and cooperates with other transcription factors (i.e., xenobiotic and oxidative response factor SKN-1) to form a network to regulate aging [51]. Therefore, a reduction in insulin signaling or activation of AMPK can induce one or both of the transcription factors DAF-16 and SKN-1, subsequently upregulating various antioxidant genes to quench the ROS level [50]. This evidence and our results seem to indicate mechanisms for the PGZ-induced lifespan benefit; that is, PGZ activated oxidative stress-related signaling, including inhibiting the IIS pathway and activating AMPK, thereby activating downstream transcription factors such as DAF-16, HSF-1, and SKN-1, ultimately resulting in an enhanced lifespan. It is worth noting that the regulation of reproductive signaling pathways by PGZ might be unrelated to its antioxidant activity because previous studies have found that long-lived *glp*-*1* mutants exhibit an increase in ROS levels rather than a decrease [52].

In summary, in this work, we found that PGZ could significantly delay the aging of wild-type worms, and the IIS, DR-like, and reproductive signaling pathways might all be involved in its regulatory mechanism. This is the first work to show that PGZ can extend the lifespan of *C*. *elegans*. This work will provide a basis for the development of PGZ as a new drug to potentially delay aging and treat aging-related diseases.

## Data Availability

The data used to support the findings of this study are included within the article and the supplementary material files.

## Supplementary Materials

Supplementary Materials Table S1: effect of PGZ on the lifespan of *C. elegans*. Table S2: the gene-specific primer sequences (qPCR).

## References

[1] López-Otín C., Blasco M. A., Partridge L., Serrano M., Kroemer G. (2013). The hallmarks of aging. *Cell*.

[2] Chen J., Ou Y., Li Y., Hu S., Shao L. W., Liu Y. (2017). Metformin extends C. elegans lifespan through lysosomal pathway. *eLife*.

[3] Wan Q. L., Zheng S. Q., Wu G. S., Luo H. R. (2013). Aspirin extends the lifespan of _Caenorhabditis elegans_ via AMPK and DAF-16/FOXO in dietary restriction pathway. *Experimental Gerontology*.

[4] Pallauf K., Rimbach G., Rupp P. M., Chin D., MA Wolf I. (2016). Resveratrol and lifespan in model organisms. *Current Medicinal Chemistry*.

[5] Ryu D., Mouchiroud L., Andreux P. A. (2016). Urolithin A induces mitophagy and prolongs lifespan in _C. elegans_ and increases muscle function in rodents. *Nature Medicine*.

[6] Chen A. L., Lum K. M., Lara-Gonzalez P. (2019). Pharmacological convergence reveals a lipid pathway that regulates _C. elegans_ lifespan. *Nature Chemical Biology*.

[7] Katsyuba E., Mottis A., Zietak M. (2018). De novo NAD^+^ synthesis enhances mitochondrial function and improves health. *Nature*.

[8] Cabreiro F., Au C., Leung K. Y. (2013). Metformin retards aging in *C. elegans* by altering microbial folate and methionine metabolism. *Cell*.

[9] Shen Z., Hinson A., Miller R. A., Garcia G. G. (2021). Cap-independent translation: a shared mechanism for lifespan extension by rapamycin, acarbose, and 17*α*-estradiol. *Aging Cell*.

[10] Seok H., Lee M., Shin E. (2019). Low-dose pioglitazone can ameliorate learning and memory impairment in a mouse model of dementia by increasing LRP1 expression in the hippocampus. *Scientific Reports*.

[11] Jafari M., Khodayari B., Felgner J., Bussel I. I., Rose M. R., Mueller L. D. (2007). Pioglitazone: an anti-diabetic compound with anti-aging properties. *Biogerontology*.

[12] Kenyon C. J. (2010). The genetics of ageing. *Nature*.

[13] Taylor R. C. (2016). Aging and the UPR(ER). *Brain Research*.

[14] Huang C., Xiong C., Kornfeld K. (2004). Measurements of age-related changes of physiological processes that predict lifespan of Caenorhabditis elegans. *Proceedings of the National Academy of Sciences of the United States of America*.

[15] Naim N., Amrit F. R. G., Ratnappan R., DelBuono N., Loose J. A., Ghazi A. (2021). Cell nonautonomous roles of NHR-49 in promoting longevity and innate immunity. *Aging Cell*.

[16] Murphy C. T., McCarroll S. A., Bargmann C. I. (2003). Genes that act downstream of DAF-16 to influence the lifespan of *Caenorhabditis elegans*. *Nature*.

[17] Nagashima T., Iino Y., Tomioka M. (2019). DAF-16/FOXO promotes taste avoidance learning independently of axonal insulin-like signaling. *PLoS Genetics*.

[18] Tullet J. M., Hertweck M., An J. H. (2008). Direct inhibition of the longevity-promoting factor SKN-1 by insulin-like signaling in *C. elegans*. *Cell*.

[19] Chiang W. C., Ching T. T., Lee H. C., Mousigian C., Hsu A. L. (2012). HSF-1 regulators DDL-1/2 link insulin-like signaling to heat-shock responses and modulation of longevity. *Cell*.

[20] Ravichandran M., Priebe S., Grigolon G. (2018). Impairing L-threonine catabolism promotes healthspan through methylglyoxal-mediated proteohormesis. *Cell Metabolism*.

[21] Dehghan E., Zhang Y., Saremi B. (2017). Hydralazine induces stress resistance and extends *C. elegans* lifespan by activating the NRF2/SKN-1 signalling pathway. *Nature Communications*.

[22] Eruslanov E., Kusmartsev S. (2010). Identification of ROS using oxidized DCFDA and flow-cytometry. *Methods in Molecular Biology*.

[23] Greer E. L., Dowlatshahi D., Banko M. R. (2007). An AMPK-FOXO pathway mediates longevity induced by a novel method of dietary restriction in *C. elegans*. *Current Biology*.

[24] Onken B., Driscoll M. (2010). Metformin induces a dietary restriction-like state and the oxidative stress response to extend C. elegans healthspan via AMPK, LKB1, and SKN-1. *PLoS One*.

[25] Lee H., Cho J. S., Lambacher N. (2008). The *Caenorhabditis elegans*AMP-activated protein kinase AAK-2 is phosphorylated by LKB1 and is required for resistance to oxidative stress and for normal motility and foraging behavior. *The Journal of Biological Chemistry*.

[26] Campisi J., Kapahi P., Lithgow G. J., Melov S., Newman J. C., Verdin E. (2019). From discoveries in ageing research to therapeutics for healthy ageing. *Nature*.

[27] Fontana L., Partridge L., Longo V. D. (2010). Extending healthy life span—from yeast to humans. *Science*.

[28] Steinbaugh M. J., Narasimhan S. D., Robida-Stubbs S. (2015). Lipid-mediated regulation of SKN-1/Nrf in response to germ cell absence. *eLife*.

[29] Hsin H., Kenyon C. (1999). Signals from the reproductive system regulate the lifespan of *C. elegans*. *Nature*.

[30] Motola D. L., Cummins C. L., Rottiers V. (2006). Identification of ligands for DAF-12 that govern dauer formation and reproduction in *C. elegans*. *Cell*.

[31] McCormick M., Chen K., Ramaswamy P., Kenyon C. (2012). New genes that extend Caenorhabditis elegans’ lifespan in response to reproductive signals. *Aging Cell*.

[32] Weinberg F., Hamanaka R., Wheaton W. W. (2010). Mitochondrial metabolism and ROS generation are essential for Kras-mediated tumorigenicity. *Proceedings of the National Academy of Sciences of the United States of America*.

[33] Yang W., Hekimi S. (2010). Two modes of mitochondrial dysfunction lead independently to lifespan extension in Caenorhabditis elegans. *Aging Cell*.

[34] Vatner S. F., Zhang J., Oydanich M., Berkman T., Naftalovich R., Vatner D. E. (2020). Healthful aging mediated by inhibition of oxidative stress. *Ageing Research Reviews*.

[35] Rea S. L., Ventura N., Johnson T. E. (2007). Relationship between mitochondrial electron transport chain dysfunction, development, and life extension in Caenorhabditis elegans. *PLoS Biology*.

[36] Larsen P. L., Clarke C. F. (2002). Extension of life-span in Caenorhabditis elegans by a diet lacking coenzyme Q. *Science*.

[37] Stiernagle T. (1999). Maintenance of C. elegans. C. elegans.

[38] Wan Q. L., Meng X., Dai W. (2021). N^6^-methyldeoxyadenine and histone methylation mediate transgenerational survival advantages induced by hormetic heat stress. *Science Advances*.

[39] Wan Q. L., Meng X., Fu X. (2019). Intermediate metabolites of the pyrimidine metabolism pathway extend the lifespan of C. elegans through regulating reproductive signals. *Aging (Albany NY)*.

[40] Wan Q. L., Fu X., Meng X. (2020). Hypotaurine promotes longevity and stress tolerance via the stress response factors DAF-16/FOXO and SKN-1/NRF2 in Caenorhabditis elegans. *Food & Function*.

[41] Wan Q. L., Fu X., Dai W. (2020). Uric acid induces stress resistance and extends the life span through activating the stress response factor DAF-16/FOXO and SKN-1/NRF2. *Aging (Albany NY)*.

[42] Chomczynski P., Sacchi N. (2006). The single-step method of RNA isolation by acid guanidinium thiocyanate-phenol-chloroform extraction: twenty-something years on. *Nature Protocols*.

[43] Dorling J. L., Martin C. K., Redman L. M. (2020). Calorie restriction for enhanced longevity: the role of novel dietary strategies in the present obesogenic environment. *Ageing Research Reviews*.

[44] Petr M. A., Tulika T., Carmona-Marin L. M., Scheibye-Knudsen M. (2020). Protecting the aging genome. *Trends in Cell Biology*.

[45] Fontana L., Partridge L. (2015). Promoting health and longevity through diet: from model organisms to humans. *Cell*.

[46] Anisimov V. N., Berstein L. M., Egormin P. A. (2008). Metformin slows down aging and extends life span of female SHR mice. *Cell Cycle*.

[47] Wu L., Zhou B., Oshiro-Rapley N. (2016). An ancient, unified mechanism for metformin growth inhibition in *C. elegans* and cancer. *Cell*.

[48] Pryor R., Norvaisas P., Marinos G. (2019). Host-microbe-drug-nutrient screen identifies bacterial effectors of metformin therapy. *Cell*.

[49] Xie M., Roy R. (2012). Increased levels of hydrogen peroxide induce a HIF-1-dependent modification of lipid metabolism in AMPK compromised *C. elegans* dauer larvae. *Cell Metabolism*.

[50] Hwang A. B., Ryu E. A., Artan M. (2014). Feedback regulation via AMPK and HIF-1 mediates ROS-dependent longevity in Caenorhabditis elegans. *Proceedings of the National Academy of Sciences of the United States of America*.

[51] Tullet J. M. A., Green J. W., Au C. (2017). The SKN-1/Nrf2 transcription factor can protect against oxidative stress and increase lifespan in C. elegans by distinct mechanisms. *Aging Cell*.

[52] Wei Y., Kenyon C. (2016). Roles for ROS and hydrogen sulfide in the longevity response to germline loss in Caenorhabditis elegans. *Proceedings of the National Academy of Sciences of the United States of America*.

