# Supplementary material for: Pioglitazone Hydrochloride Extends the Lifespan of Caenorhabditis elegans by Activating DAF-16/FOXO- and SKN-1/NRF2-Related Signaling Pathways
Source: Oxid Med Cell Longev. 2022 May 29;2022:8496063. doi: 10.1155/2022/8496063 (PMC9168093; doi:10.1155/2022/8496063)
Supplement: Supplementary Materials — Table S1: effect of PGZ on the lifespan of C. elegans. Table S2: the gene-specific primer sequences (qPCR). [file 8496063.f1.docx]

**Pioglitazone Hydrochloride Extends the Lifespan of *Caenorhabditis elegans* by Activating DAF-16/FOXO- and SKN-1/NRF2-related Signaling Pathways**

Wenjuan Jia ^1, 2, #^, Chongyang Wang ^3, #^, Jingming Zheng ^4^, Yimin Li ^4^, Caixian Yang ^2^, Qin-Li Wan ^4, *^, Jie Shen ^1, 5, *^

^1^Department of Endocrinology and Metabolism, The Third Affiliated Hospital, Southern Medical University, Guangzhou 510630, China

^2^Department of Endocrinology, Sixth Affiliated Hospital of Guangzhou Medical University, Qingyuan People’s Hospital, Qingyuan 511518, China

^3^Zhuhai Precision Medical Center, Zhuhai People’s Hospital (Zhuhai Hospital Affiliated with Jinan University), Jinan University, Guangzhou 510632, China

^4^Department of pathogen biology, school of medicine, Jinan University, Guangzhou 510632, China

^5^Institute and Department of Endocrinology and Metabolism, Shunde Hospital，Southern Medical University (The First People's Hospital of Shunde), Foshan 528399, China

^#^ Wenjuan Jia and Chongyang Wang contributed equally to this work.

* Correspondence should be addressed to Jie Shen; [sjiesy@smu.edu.cn](mailto:sjiesy@smu.edu.cn) and Qin-Li Wan; wanqinli@hotmail.com

**Table S1** Effect of PGZ on the lifespan of C. elegans

| **Figure** | **Strains** | **Treatments** | **Mean**  **Lifespan ± SEM**  **(days)** | ***P* value**  **VS**  **Control** | **%**  **Change in mean lifespan** | **N** |
| --- | --- | --- | --- | --- | --- | --- |
| **N2(WT)** | |  |  |  |  |  |
| **Figure 1** | EXP. 1 | 20 ℃ / Control | 20.766±0.327 |  |  | 94 |
|  | EXP. 1 | 20 ℃ / 0.1 mM PGZ | 22.694±0.296 | <0.0001 | 9.284 | 111 |
|  | EXP. 1 | 20 ℃ / 0.5 mM PGZ | 24.457±0.333 | <0.0001 | 17.77 | 138 |
|  | EXP. 1 | 20 ℃ / 2 mM PGZ | 20.986±0.491 | 0.122 | 1.059 | 74 |
|  | EXP. 2 | 20 ℃ / Control | 21.787±0.319 |  |  | 94 |
|  | EXP. 2 | 20 ℃ / 0.1 mM PGZ | 23.850±0.375 | <0.0001 | 9.469 | 80 |
|  | EXP. 2 | 20 ℃ / 0.5 mM PGZ | 24.167±0.405 | <0.0001 | 10.923 | 90 |
|  | EXP. 3 | 20 ℃ / Control | 20.184±0.413 |  |  |  |
|  | EXP. 3 | 20 ℃ / 0.1 mM PGZ | 21.000±0.310 | 0.600 | 3.678 | 90 |
|  | EXP. 3 | 20 ℃ / 0.5 mM PGZ | 23.013±0.373 | <0.0001 | 12.754 | 80 |
|  | EXP. 4 | 20 ℃ / Control | 20.906±0.309 |  |  | 117 |
|  | EXP. 4 | 20 ℃ / 0.1 mM PGZ | 22.695±0.286 | <0.0001 | 8.166 | 105 |
|  | EXP. 4 | 20 ℃ / 0.5 mM PGZ | 24.553±0.309 | <0.0001 | 16.648 | 114 |
|  | EXP. 4 | 20 ℃ / 2 mM PGZ | 22.738±0.335 | <0.0001 | 8.363 | 107 |
|  | | | | | | |
| **PS3551 *hsf-1(sy441) I*** | | | | | | |
| **Figure 3A** | EXP. 1 | 20 ℃ / Control | 20.351±0.347 |  |  | 131 |
|  | EXP. 1 | 20 ℃ / 0.5 mM PGZ | 19.376±0.426 | 0.101 | # | 93 |
|  | EXP. 2 | 20 ℃ / Control | 21.571±0.376 |  |  | 140 |
|  | EXP. 2 | 20 ℃ / 0.5 mM PGZ | 21.791±0.37 | 0.681 | # | 158 |
|  | EXP. 3 | 20 ℃ / Control | 20.992±0.360 |  |  | 127 |
|  | EXP. 3 | 20 ℃ / 0.5 mM PGZ | 21.503±0.303 | 0.705 | # | 153 |
|  |  |  |  |  |  |  |
| ***MQ887 isp-1(qm150) IV*** | | | | | | |
| **Figure 5A** | EXP. 1 | 20 ℃ / Control | 30.216±0.515 |  |  | 88 |
|  | EXP. 1 | 20 ℃ / 0.5 mM PGZ | 33.604±0.512 | < 0.0001 | 11.213 | 96 |
|  | EXP. 2 | 20 ℃ / Control | 28.023±0.445 |  |  | 128 |
|  | EXP. 2 | 20 ℃ / 0.5 mM PGZ | 31.757±0.458 | < 0.0001 | 13.325 | 107 |
|  | EXP. 3 | 20 ℃ / Control | 28.414±0.523 |  |  | 111 |
|  | EXP. 3 | 20 ℃ / 0.5 mM PGZ | 32.774±0.425 | < 0.0001 | 15.345 | 124 |
|  | | | | | | |
| ***CF1903 glp-1(e2144) III*** | | | | | | |
| **Figure 4C** | EXP. 1 | 20 ℃ / Control | 30.878±0.827 |  |  | 90 |
|  | EXP. 1 | 20 ℃ / 0.5 mM PGZ | 31.608±0.692 | 0.141 | # | 120 |
|  | EXP. 2 | 20 ℃ / Control | 29.222±0.525 |  |  | 153 |
|  | EXP. 2 | 20 ℃ / 0.5 mM PGZ | 27.129±1.984 | 0.195 | # | 70 |
|  | EXP. 3 | 20 ℃ / Control | 30.712±0.627 |  |  | 146 |
|  | EXP. 3 | 20 ℃ / 0.5 mM PGZ | 29.928±0.922 | 0.457 | # | 69 |
|  |  |  |  |  |  |  |
| ***CF1038 daf-16(mu86) I*** | | | | | | |
| **Figure 2D** | EXP. 1 | 20 ℃ / Control | 19.967±0.293 |  |  | 122 |
|  | EXP. 1 | 20 ℃ / 0.5 mM PGZ | 18.865±0.449 | 0.063 | # | 74 |
|  | EXP. 2 | 20 ℃ / Control | 19.574±0.330 |  |  | 108 |
|  | EXP. 2 | 20 ℃ / 0.5 mM PGZ | 19.211±0.530 | 0.220 | # | 76 |
|  | EXP. 3 | 20 ℃ / Control | 20.309±0.486 |  |  | 81 |
|  | EXP. 3 | 20 ℃ / 0.5 mM PGZ | 20.355±0.510 | 0.657 | # | 76 |
|  |  |  |  |  |  |  |
| ***DA1116 eat-2(ad1116) II*** | | | | | | |
| **Figure 4B** | EXP. 1 | 20 ℃ / Control | 26.078±0.602 |  |  | 77 |
|  | EXP. 1 | 20 ℃ / 0.5 mM PGZ | 26.950±0.595 | 0.063 | # | 101 |
|  | EXP. 2 | 20 ℃ / Control | 26.622±0.569 |  |  | 90 |
|  | EXP. 2 | 20 ℃ / 0.5 mM PGZ | 25.395±0.443 | 0.051 | # | 147 |
|  | EXP. 3 | 20 ℃ / Control | 28.302±0.569 |  |  | 53 |
|  | EXP. 3 | 20 ℃ / 0.5 mM PGZ | 27.042±0.483 | 0.399 | # | 120 |
|  |  |  |  |  |  |  |
| ***CB4876 clk-1(e2519) III*** | | | | | | |
| **Figure 5B** | EXP. 1 | 20 ℃ / Control | 29.159±0.935 |  |  | 82 |
|  | EXP. 1 | 20 ℃ / 0.5 mM PGZ | 33.854±0.834 | 0.002 | 13.868 | 65 |
|  | EXP. 2 | 20 ℃ / Control | 29.956±0.608 |  |  | 114 |
|  | EXP. 2 | 20 ℃ / 0.5 mM PGZ | 32.676±0.846 | <0.0001 | 9.07 | 71 |
|  | EXP. 3 | 20 ℃ / Control | 28.589±0.363 |  |  | 190 |
|  | EXP. 3 | 20 ℃ / 0.5 mM PGZ | 30.770±0.425 | <0.0001 | 24.269 | 135 |
|  | | | | | | |
| ***EU1 skn-1(zu67) IV*** | | | | | | |
| **Figure 3B** | EXP. 1 | 20 ℃ / Control | 19.494±0.564 |  |  | 89 |
|  | EXP. 1 | 20 ℃ / 0.5 mM PGZ | 19.620±0.447 | 0.187 | # | 79 |
|  | EXP. 2 | 20 ℃ / Control | 20.730±0.486 |  |  | 89 |
|  | EXP. 2 | 20 ℃ / 0.5 mM PGZ | 20.444±0.363 | 0.232 | # | 133 |
|  | EXP. 3 | 20 ℃ / Control | 19.783±0.598 |  |  | 83 |
|  | EXP. 3 | 20 ℃ / 0.5 mM PGZ | 18.838±0.487 | 0.029 | -5.016 | 80 |
|  |  |  |  |  |  |  |
| ***CB1370 daf-2(e1370) III*** | | | | | | |
| **Figure 2B** | EXP. 1 | 20 ℃ / Control | 48.651±0.811 |  |  | 63 |
|  | EXP. 1 | 20 ℃ / 0.5 mM PGZ | 47.553±0.510 | 0.011 | # | 132 |
|  | EXP. 2 | 20 ℃ / Control | 47.708±0.617 |  |  | 106 |
|  | EXP. 2 | 20 ℃ / 0.5 mM PGZ | 47.935±0.415 | 0.717 | # | 186 |
|  | EXP. 3 | 20 ℃ / Control | 49.295±0.844 |  |  | 44 |
|  | EXP. 3 | 20 ℃ / 0.5 mM PGZ | 49.520±0.385 | 0.108 | # | 152 |
|  |  |  |  |  |  |  |
| ***RB754 aak-2(ok524) X*** | | | | | | |
| **Figure 4A** | EXP. 1 | 20 ℃ / Control | 20.758±0.390 |  |  | 91 |
|  | EXP. 1 | 20 ℃ / 0.5 mM PGZ | 20.772±0.325 | 0.730 | # | 123 |
|  | EXP. 2 | 20 ℃ / Control | 20.511±0.306 |  |  | 88 |
|  | EXP. 2 | 20 ℃ / 0.5 mM PGZ | 19.780±0.304 | 0.012 | -3.564 | 91 |
|  | EXP. 3 | 20 ℃ / Control | 20.781±0.273 |  |  | 73 |
|  | EXP. 3 | 20 ℃ / 0.5 mM PGZ | 19.833±0.328 | 0.021 | -4.562 | 54 |
|  | | | | | | |
| ***GR1310 akt-1(mg144) V*** | | | | | | |
| **Figure 2C** | EXP. 1 | 20 ℃ / Control | 22.425±0.312 |  |  | 80 |
|  | EXP. 1 | 20 ℃ / 0.5 mM PGZ | 21.857±0.394 | 0.136 | # | 49 |
|  | EXP. 2 | 20 ℃ / Control | 20.871±0.514 |  |  | 62 |
|  | EXP. 2 | 20 ℃ / 0.5 mM PGZ | 20.859±0.471 | 0.671 | # | 171 |
|  | EXP. 3 | 20 ℃ / Control | 22.726±0.411 |  |  | 73 |
|  | EXP. 3 | 20 ℃ / 0.5 mM PGZ | 21.736±0.401 | 0.600 | # | 87 |
|  | | | | | | |
| ***AA86 daf-12(rh61rh411) X*** | | | | | | |
| **Figure 4D** | EXP. 1 | 20 ℃ / Control | 21.635±0.617 |  |  | 74 |
|  | EXP. 1 | 20 ℃ / 0.5 mM PGZ | 22.526±0.418 | 0.732 | # | 95 |
|  | EXP. 2 | 20 ℃ / Control | 21.961±0.708 |  |  | 51 |
|  | EXP. 2 | 20 ℃ / 0.5 mM PGZ | 23.193±0.432 | 0.107 | # | 83 |
|  | EXP. 3 | 20 ℃ / Control | 21.322±0.361 |  |  | 121 |
|  | EXP. 3 | 20 ℃ / 0.5 mM PGZ | 22.064±0.330 | 0.343 | # | 110 |
|  |  |  |  |  |  |  |
| ***VC870 nhr-49(gk405) I*** | | |  |  |  |  |
| **Figure 2A** | EXP. 1 | 20 ℃ / Control | 20.769±0.398 |  |  | 104 |
|  | EXP. 1 | 20 ℃ / 0.5 mM PGZ | 19.926±0.429 | 0.042 | -4.05 | 68 |
|  | EXP. 2 | 20 ℃ / Control | 20.664±0.485 |  |  | 116 |
|  | EXP. 2 | 20 ℃ / 0.5 mM PGZ | 19.900±0.566 | 0.206 | # | 60 |
|  | EXP. 3 | 20 ℃ / Control | 19.213±0.441 |  |  | 75 |
|  | EXP. 3 | 20 ℃ / 0.5 mM PGZ | 18.379±0.338 | 0.047 | # | 95 |

**Table S1** Survival analyses were conducted using the Kaplan-Meier method. *P* values were calculated by log-rank test for individual experiments. All statistical were performed using SPSS package. “N” displayed the number of dead worms. “#”: no calculate (because p > 0.05).

**Table S2: The gene-specific primers sequences (qPCR)**

| **Primer sequences used for quantitative PCR (5′→3′):** | | |
| --- | --- | --- |
| **Gene** | **Forward primer sequence** | **Reverse primer sequence** |
| *cdc-42* | CTGCTGGACAGGAAGATTACG | CTCGGACATTCTCGAATGAAG |
| *sod-1* | CGTAGGCGATCTAGGAAATGTG | AACAACCATAGATCGGCCAACG |
| *sod-3* | AGCATCATGCCACCTACGTGA | AGCATCATGCCACCTACGTGA |
| *ctl-1* | GACGTATCCAAAACCCCAAGTG | TTGGCATGAACGACACGCTC |
| *ctl-2* | TTCCGATCGAGGACTCCCAG | CTTCACTCCTTGAGTTGGCTTG |
| *dod-3* | CGTATATGGACCCAGCTAATG | ATGAACACCGGCTCATTC |
| *fard-1* | GGGTTTTTGGGAAAGGTGAT | CCACCGATTGCTTTCAATTT |
| *lips-17* | ATCTGTTGCTGGAGCCAATCG | TATCCAACTTTATCGTCTCC |
| *cdr-6* | TCGGGCTTCTCGGTTTACC | CAGCTTTGACCAGAGGAACCA |
| *skn-1* | TGGAGTGTCGTCCATATTCATCT | TGAGGTGTTGGACGATGGTG |
| *gst-4* | TCCGTCAATTCACTTCTTCCG | AAGAAATCATCACGGGCTGG |
